# Effectiveness of a hand-held fan for breathlessness: a randomised phase II trial

**DOI:** 10.1186/1472-684X-9-22

**Published:** 2010-10-19

**Authors:** Claudia Bausewein, Sara Booth, Marjolein Gysels, Robert Kühnbach, Irene J Higginson

**Affiliations:** 1 King's College London, Department of Palliative Care, Policy & Rehabilitation, Cicely Saunders Institute, London, UK; 2 Palliative Care Team, Addenbrooke's Hospital, Cambridge, UK; 3 Barcelona Centre for International Health Research, Universitat de Barcelona, Barcelona, Spain; 4 Interdisciplinary Centre for Palliative Medicine, Munich University Hospital, Munich, Germany

## Abstract

**Background:**

Breathlessness is common and distressing in advanced disease. This phase II study aimed to determine the use and acceptance of a hand-held fan (HHF) to relieve breathlessness, to test the effectiveness of the HHF and to evaluate the recruitment into the study.

**Methods:**

RCT embedded within a longitudinal study testing a HHF over time compared to a wristband. Patients were included in the longitudinal study when suffering from breathlessness due to advanced cancer or COPD III/IV and could opt in the RCT. Primary outcome was use of the HHF and the wristband after two months. Secondary outcomes were recruitment into the trial and change of breathlessness severity after two months, measured on the modified Borg scale. Baseline data were collected in a personal interview and follow-up data by monthly postal questionnaires.

**Results:**

109 patients were recruited in the longitudinal study of which 70 patients (64%) participated in the RCT. Non-participants had statistically significant less breathlessness (Borg mean 2.6 (SD 1.48) versus 3.7 (SD 1.83); p = 0.003) and a better functional status (Karnofsky status mean 61.9 (SD 11.2) versus 66.7 (SD 11.0); p = 0.03). Attrition due to drop out or death was high in both groups. After two months, about half of the patients used the HHF but only 20% the wristband without a statistical difference (Fisher's exact test p = 0.2). 9/16 patients judged the HHF as helpful after two months and 4/5 patients the wristband. There was no difference in mean breathlessness change scores between the HHF (Borg change score: mean 0.6 (SD 2.10)) and the wristband (mean 0.8 (SD 2.67)) after two months (p = 0.90).

**Conclusions:**

Symptom burden and low functional status did not restrain patients from participation in the study. Finding a control for a visible intervention is challenging and needs careful consideration to what is acceptable to patients. The preliminary evidence of effectiveness of the HHF could not be proved. Patients often stopped using the HHF but a small group seemed to benefit which was not necessarily related to a relief in breathlessness. Therefore, more work is necessary on selecting and identifying those who might benefit from the HHF.

**Trial registration:**

ClinicalTrials.gov Identifier: NCT01123902

## Background

Breathlessness is a common and distressing symptom in advanced disease [1] which is still poorly understood and not managed satisfactorily [2]. Many non-pharmacological interventions are available which may complement pharmacological interventions in the management of breathlessness [3]. However, the evidence is scarce for some of these interventions, e.g. a hand-held fan (HHF). This simple and cheap device is easy to use and it is one of the few interventions that can be used by the patients independent of any clinician or setting. A HHF produces a flow of air which may alter ventilation when directed to the face, nasal mucosa, or pharynx [4] but the exact mechanism of this effect is unclear. It has been used successfully to reduce breathlessness in healthy participants where breathlessness was induced through inspiratory resistive load [4]. Two studies tested the HHF in patients [5,6]. A small pilot study in six COPD patients did not show sufficient improvement of breathlessness [5] but an adequately powered crossover trial in 50 palliative care patients showed a significant improvement in breathlessness [6]. This latter study tested the HHF directed five minutes to the face or leg and crossed over to the other treatment with a 10 minutes washout period. As the results of this study were promising, the HHF should be tested in a real world setting where patients use it over time. However, before setting up an adequately powered RCT we felt that a number of questions needed to be addressed about the feasibility of such a study. Therefore, we ran an exploratory phase II study to test a HHF for breathlessness which aimed to evaluate the use and acceptance of the intervention and the control, the potential effectiveness of the HHF, the recruitment into the study, and collecting data for calculating a sample size.

## Methods

### Design

As we wanted to assess how the HHF worked over time, we embedded this RCT in a longitudinal cohort study which aimed to describe the course of breathlessness over time [7].

### Setting and recruitment

Recruitment to the longitudinal study took place from the oncology and/or respiratory departments in three major hospitals (one tertiary respiratory hospital), a hospice home care service and two respiratory practices in Munich, Germany. All recruitment sites screened patients regularly for inclusion criteria, and patients in the respiratory hospital were screened at regular weekly visits. The longitudinal and RCT components were explained in more detail to patients interested in the study, and an information leaflet was provided. There was one consent form for both studies but patients were given the option to participate only in the longitudinal study and not in the RCT. Reasons for refusal to participate in the RCT were recorded. Data were collected from June 2006 to November 2007.

### Patients

Patients were included if they reported breathlessness which had an impact on their daily life and were suffering from one of the following conditions:

• Advanced malignant disease (primary lung cancer or secondary lung metastases/lung involvement due to cancer).

• COPD stage III (severe) and IV (very severe) according to GOLD criteria[8].

Patients were excluded if unable to provide informed consent, too ill to be interviewed and not fluent or literate in German. There were no additional exclusion criteria for the RCT.

### Randomisation and allocation concealment

A computer-generated random number list was produced using stratified randomisation with blocks of six to ensure exactly equal treatment numbers at certain equally spaced points in the sequence of patient assignments [9]. COPD and cancer were determined as strata and a separate randomisation list was prepared for each stratum. After consent and baseline interview, randomisation was conducted using an independent individual who opened prepared and sealed envelopes.

### Intervention and control

The intervention to be tested was a HHF directed to the area of the face innervated by the second and third trigeminal nerve branches. At the first contact, patients received a HHF and the researcher demonstrated how to use the HHF showing the appropriate area around the central part of the face, the sides of the nose and above the upper lip. The HHF had three soft rotor blades and an unfoldable rotor unit. Patients also received an information leaflet with a picture of a HHF, explanations and instructions for the use of the HHF.

A wristband was chosen as control under the assumption that distraction could serve as a placebo and was more realistic than directing the fan towards the leg as in a previous study [6]. At the first contact, patients received a plastic wristband labelled "breathe easy". They were instructed to wear the wristband continually and pull it regularly at short intervals when breathless or during breathlessness attacks. Patients in this group received a similar information leaflet as the HHF group but for the use of the wristband.

Both patient groups received standard care supplied by local services including general practitioners, district nurses, specialist respiratory medicine or oncology, and potentially palliative care.

### Outcomes

The primary outcome for this study was the use of the HHF and the wristband after two months. Secondary outcomes were helpfulness of the HHF and the wristband after two months, change of breathlessness severity between baseline and month two, and uptake into the trial (proportion of patients from the longitudinal study participating in the RCT). We were also interested in patients' experiences using the HHF and the wristband.

To assess patients' use, helpfulness and experiences with the HHF and the wristband over time, specific questions were asked monthly over six months as part of the questionnaires in the longitudinal study (see Table 1).

**Table 1:** Questions on use and experiences with hand-held fan or wristband

Question	Answer options
1. How often do you use the hand-held fan/wristband?	daily, occasionally, not at all
2. Do you find the hand-held fan/wristband helpful?	yes, no, don't know
3. What are your experiences with the hand-held fan/wristband?	Free text
4. Do you have any comments regarding the hand-held fan/wristband?	Free text

The modified Borg scale, a categorical scale with ratio properties, was used to assess the average severity of breathlessness over the last 24 hours [10-12]. Borg scores range from 0 (no breathlessness) to 10 (maximum breathlessness). The scale is widely used in respiratory medicine and has been used with cancer patients [13].

### Timing of data collection

Data collection used an initial face to face interview; patients were followed by monthly postal questionnaires for six months or until the patient died.

### Analysis

Demographic and clinical characteristics of patients taking part in the RCT and those refusing to participate were compared, using unpaired t-tests for continuous and χ2-tests for categorical variables.

We compared the proportion of patients being alive who reported using the HHF or the wristband after two months, and also the helpfulness of both, using Fisher's exact test for small frequencies. Patients' comments on their experiences with the HHF and the wristband were collated and grouped in different categories (positive effects, negative effects or disbeliefs, not used and no answer).

To compare the effect of the HHF and the wristband on breathlessness, regression analysis was performed on breathlessness change scores after two months and adjusted for baseline differences. Analysis was on the basis of intention to treat where people were assigned to original groups irrespective of the use of the HHF or wristband. The significance level was set at p < 0.05. The data was analysed using the software package STATA IC10.

We calculated the effect size d of the HHF with the following formula

$$d=\frac{M1-M2}{\sqrt{(SD1^{2}+SD2^{2})/2}}$$

with M1 as the mean of the intervention group and M2 the mean of the control group [14]. Cohen (1988) defined effect sizes as "small, *d *= .2," "medium, *d *= .5," and "large, *d *= .8" [14]. With the information from this study we estimated a sample size for a bigger trial at p < 0.05 and a power of 80%.

### Sample size calculation

As this was a phase II trial we did not estimate a prior sample size. Instead we aimed to estimate an effect size for a phase III trial. The sample size for the longitudinal study has been set at 85 to show a difference of 1.0 on the Borg scale between the two groups with a standard deviation of 2.1.

We estimated that about 30 patients in each arm would give sufficient information on the feasibility of the intervention, and allow calculation of an effect size.

Ethics approval was obtained from the College Research Ethics Committee at King's College London (CREC number 05/06-69) and from the Research Ethics Committee of the University of Munich (number 079-06).

## Results

**Figure 1:**
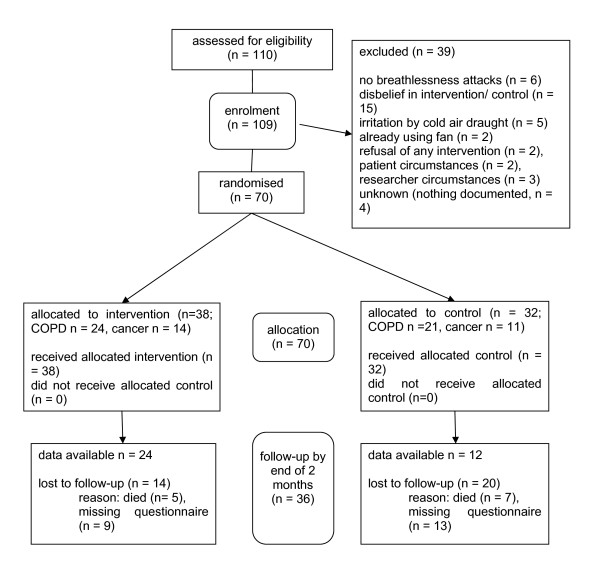
**CONSORT diagram showing the flow of patients through the study**.

109 patients were recruited in the longitudinal study. 39/109 enrolled patients declined to participate in the RCT. Main reasons for refusal were lack of belief in the intervention or control (n = 15), not suffering from dyspnoea attacks (n = 6) and feeling irritated by cold air draught (n = 5). Of the remaining seventy patients, 38 were randomised to the HHF (24 COPD and 14 cancer patients) and 32 to the use of the wristband (21 COPD and 11 cancer patients) (see Figure 1 CONSORT diagram). Main reasons for loss of follow-up were non-return of questionnaires.

### Demographic and clinical characteristics

Participants and non-participants in the RCT were similar regarding age and gender but more cancer than COPD patients refused to take part in the RCT (see Table 2). Non-participants had less severe breathlessness (t = -3.09, p = 0.003) and a better functional status (Karnofsky performance status, KPS) compared to those participating (t = 2.16, p = 0.03).

**Table 2:** Demographics of 70 patients taking part in the RCT and 39 patients excluded from the RCT

	RCT group (n = 70)	Not included in RCT (n = 39)	Comparison between groups
**Age**	65.6 (8.80)	62.7 (10.21)	t = -1.56, p = 0.12
**gender (m/f)**	m 36/f 34	m 18/f 21	χ2 = 0.28, p = 0.6
**COPD (n = 60)** **Cancer (n = 49)**	45 25	15 24	χ2 = 6.75, p = 0.009
**Borg scale**	3.7 (1.83)	2.6 (1.48)	t = -3.09, p = 0.003
**KPS**	61.9 (11.2)	66.7 (11.0)	t = 2.16, p = 0.03

Of those patients entering the RCT, the demographic and clinical variables were similar in the HHF and the wristband group regarding age, gender, diagnoses, breathlessness and use of oxygen (see Table 3).

**Table 3:** Demographics of 70 patients taking part in the RCT

	Fan (n = 38)	Wristband (n = 32)	Comparison between HHF and wristband
**Age**	64.5 (9.88)	66.6 (7.79)	t = 0.997, p = 0.32
**gender (m/f)**	m 19/f 19	m 17/f 15	χ2 = 0.07, p = 0.79
**COPD** **Cancer**	24 14	21 11	χ2 = 0.33, p = 0.57
**Borg scale**	4.0 (1.86)	3.3 (1.77)	t = 1.42, p = 0.16*
**KPS**	62.4 (10.5)	61.2 (12.1)	t = 0.41, p = 0.68

* Mann Whitney U not significant

### Attrition

The proportion of attrition and missing data was considerable in both groups. After two months, 13% of 38 patients (5 patients, 4 cancer) have died in the intervention and 22% of 32 patients in the control group (7 patients, 6 cancer). Overall, 29% of 38 patients (11 patients; 9 cancer) died in the intervention and 41% of 32 patients (13 patients, 10 cancer) in the control group during the six months of the study. Additional missing data were mainly related to intermittent missing questionnaires due to deterioration and questionnaire fatigue. Overall, missing data was higher in the control group (up to 60%) than in the intervention group (up to 50%). We did not impute missing data at this time as our main intention was the feasibility of such a study and also exploring the nature of missing data.

### Did participants use the hand-held fan and the wristband and was it helpful?

**Figure 2:**
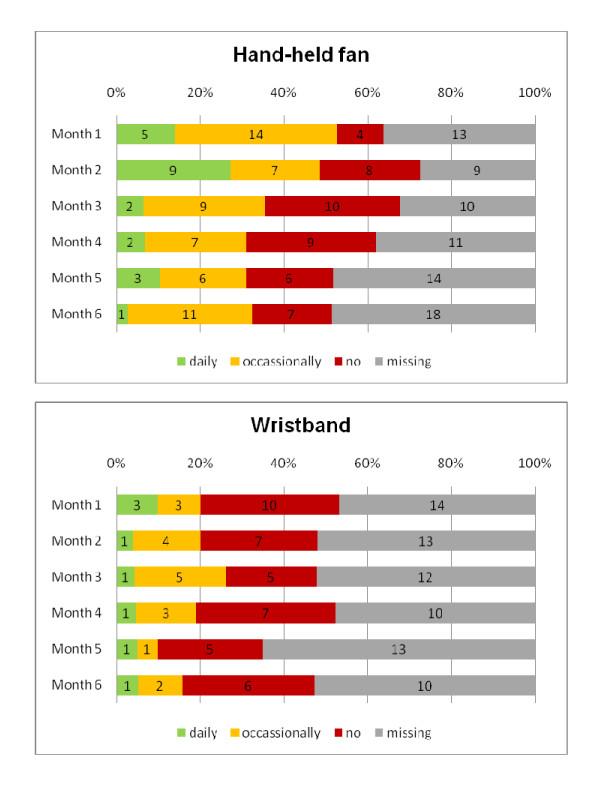
**Use of HHF (n = 38) and wristband (n = 32) over time**. (note: numbers in bars are absolute numbers)

After two months, 16/33 (48%) patients being alive used the HHF, nine daily and seven occasionally. In the wristband group, 5/25 (20%) patients being alive used it, one patient daily and four patients occasionally (see Figure 2). There was no statistical difference between the two groups (Fisher's exact p = 0.2).

The overall use over time varied in both groups. The number of patients using the HHF regularly dropped after two months considerably whereas the proportion of patients using the wristband was low from the first month on (see Figure 2).

At month two, 23 patients reported on the helpfulness of the HHF with nine considering it as helpful and 14 not. Of the 11 patients commenting on the helpfulness of the wristband four considered it to be helpful and seven not. Those not using the HHF or the wristband judged it as unhelpful. The difference in outcome was not statistically significant (Fishers exact test, p = 0.5).

### Effect of hand-held fan and wristband on breathlessness

After two months, data for change scores was available from 24 patients in the intervention and 12 patients in the control group. The mean change score for the intervention group was 0.6 (SD 2.10) and 0.8 (SD 2.67) for the control group without a statistically significant difference after adjusting for baseline Borg scores (t = 0.13, p = 0.90).

### What experiences did participants have using the hand-held fan or the wristband?

All comments of each patient were summarized to achieve an overall judgement of the experiences. 13/38 patients randomised to the use of the HHF judged their experiences as positive at some point during the study. They commented the HHF as "very good, use it regularly" (COPD patient), "very helpful, don't want to miss it anymore, makes life much easier" (COPD patient) or "helps breathing easier if you put it directly in front of mouth and nose" (COPD patient). Seven patients expressed negative experiences regarding the HHF reporting "it makes me nervous" (COPD patient) or "it is more like a toy, but doesn't help" (COPD patient). Two patients reported that the HHF was defective. One patient was very sensitive to the air draught produced by the HHF. Eight patients did not comment further on their experiences. Patients testing the wristband reported the following: 5/32 patients expressed positive experiences such as "it is reassuring to wear it" (COPD patient) or "I rely on it" (cancer patient). Four patients were rather negative pointing out that it is "foolish" (cancer patient) or "not helpful" (COPD patient). Two patients reported skin irritations and two patients did not use it of whom one complained that the wristband did not fit. Two patients had mixed experiences and seven patients did not comment further on their experiences.

### Calculation of effect size and sample size

Taking data from the second month with 12 patients in each group, we should have been able to detect an effect size of 2.5 (power of 80%, 5% significance level). However, calculating the actual effect size with the results of the second months gave us a value of -0.08.

## Discussion

The results of this phase II study give helpful insights regarding recruitment, use and acceptance of a non-pharmacological intervention, and selection of a control, for conducting a longitudinal RCT to test a non-pharmacological intervention in a palliative care population.

### Recruitment into the study

First, a two step approach was chosen to recruit patients into the RCT. All participants in the longitudinal study were invited to enter the RCT. However, only two thirds of patients took part in the RCT and a considerable number of patients opted out of the trial. Reasons for non-participation varied from irritation by cold air to lack of belief in the intervention or the control. It can only be surmised why patients believed neither in the intervention nor the control. A HHF is a simple device and some participants judged it more as a childish gadget rather than a tool that could relieve their breathlessness. Or some might expect a more technical device especially when already on long-term oxygen.

A strength of this study is the provision of baseline data of non-participants to allow comparisons with those participating in the trial. It is noteworthy that patients who refused participation in the RCT had less breathlessness and a better functional status compared to those who participated. Hence, RCT-participants were more ill than their counterparts. It can be assumed that those participating were more motivated to try something to relieve their breathlessness. Patients' physical status and symptom burden are a regular argument for gate keeping in palliative care research [15]. Data from this study further disproves the widely held opinion that patients towards the end of life may be too ill to take part in trials in palliative care.

### Acceptance of intervention and feasibility of control

Collecting data on use and experiences of patients with the intervention and the control allowed us to assess the acceptance and feasibility of both. Overall use of the HHF and the wristband was not as high as expected. In the second month, about 40% of the patients randomised to the HHF actually used it and of those about half found it helpful. The benefit from the HHF was not necessarily related to a relief in breathlessness severity. Thus, further research should identify those patients who benefit from the HHF and explore reasons for the beneficial effect. However, for most patients neither the HHF nor the wristband was popular. This is reflected in the considerable number of patients discontinuing either the intervention or the control or dropping out from the study.

For many patients who were invited to the RCT or even for those who took part, the wristband did not seem an acceptable control. This is also reflected in the comments of some patients after randomisation to the wristband that they would have preferred the HHF as they did not believe in the wristband. Compliance with the control was low from the beginning. Patients' preferences not only influence participation in randomised trials [16] but are also associated with treatment effects [17]. There is some evidence that preferences can modify treatment outcomes (especially in the preferred treatment group) but it has also been shown that participants allocated to their undesired treatment were less likely to be lost to follow-up [17]. This finding is in contrast to the observations in this study.

Finding a suitable control for a non-pharmacological intervention such as the HHF is a challenge. It has been suggested that the choice of a control should be supported by a systematic review [18]. A Cochrane Review on non-pharmacological interventions to relieve breathlessness was conducted in advance [3]. The two previous studies testing the HHF either added the fan to nasal cannulae with the flow of oxygen or directed the fan to the patient's leg [5,6]. Both options may be useful in a more controlled setting but would not be applicable in a long term outpatient setting as in this trial. The combination with oxygen would have been a co-intervention and not all patients were on oxygen. Also, repeated direction of the HHF towards the leg did neither seem practical nor plausible for patients. Several other options have been considered for the control. One was, to change the direction of the rotation of the blades of the HHF to reduce the air flow but this would have been technically difficult. Another one was to show the patients sham-acupressure points but it was felt that patients should be given a device and not only shown a procedure. As cognitive-behavioural strategies play an important role in modulating the perception of breathlessness [19], distraction in the form of pulling a wristband when breathless seemed to be an appropriate alternative.

### Effectiveness of hand-held fan

In contrast to previous studies on the use of HHF to relieve breathlessness [5,6], this study did not show a benefit of the HHF. Baltzan demonstrated transient relief of breathlessness from a fan blowing onto the face in a small group of COPD patients during an exercise test [5]. In a different study, Galbraith tested the effect of the HHF in a cross-over RCT in patients with advanced disease [6]. This study showed a benefit of the HHF in relieving breathlessness. Both studies used the fan only over a short time period, Baltzan over three days and Galbraith only once. As both studies lacked follow-up, the long-term effects of the HHF were unclear. Participants in our trial did not use the HHF or the wristband consistently over time and potential reasons for this have been discussed above. It could also be surmised whether there are cultural differences in Germany regarding the acceptance of such an intervention and control.

### Collecting data for calculating effect size and sample size

A further aim of this study was to collect data for calculating an effect size to be able to calculate a sample size for a larger trial. The effect size that we derived from our data was minimal (-0.08). This low effect size and the low continued use of the HHF question the value of the HHF at least in COPD and cancer patients. This pragmatic phase II trial needs replication as it is in contrast to earlier work.

### Limitations

Ideally, the intervention in an RCT should be blinded as otherwise the results may be distorted if patients and/or investigator know which treatment is being used [9]. However, non-blinding would have meant that patients would have been more likely to report a benefit - and researchers too. As we didn't find a benefit it is more likely/impossible that the result would be found because of non-blinding. Indeed it makes our negative result more likely.

Blinding of a non-pharmacological intervention such as the HHF is almost impossible in many cases. If blinding is not feasible one compromise could be to blind the evaluation and/or the analysis. The first was also not feasible in this study as patients filled in the postal questionnaires themselves without a researcher. The second would have been a potential approach which was not used in this study but should be considered for future trials in non-pharmacological interventions.

A further limitation is the small number of patients providing data for analysis and consequently the loss of power. Our main aim was to collect information about the feasibility of testing a HHF and to a lesser extent to evaluate the effectiveness of the intervention. Nevertheless, as discussed before, attrition and low compliance of adherence to the intervention and the control were considerable and not expected. It was tried to increase compliance by providing an information leaflet and instructions on how to use the device but reminders and further instructions during the trial were forgone as the use of the devices over time was one of the outcomes.

## Conclusions

This study does not allow a conclusion about the effectiveness of a HHF to relieve breathlessness. However, it seems that a small group of patients is getting benefit from a HHF although this may not be related to reduction of breathlessness severity. As the HHF is a cheap and simple device a trial with an individual patient should be considered. Further research should focus not only on the effectiveness of a HHF but also on predictors for whom it may be helpful.

Findings from this study also support the benefit of running phase II trials before setting up larger RCTs to test recruitment and, especially when non-pharmacological interventions are tested, the feasibility of the intervention and the control. Selecting a control for a non-pharmacological intervention is challenging and needs careful consideration to what is acceptable to patients. Symptom burden and low functional status did not restrain patients from participation in the study which emphasises the importance of giving patients the choice whether they want to take part in research or not. Timing of outcome measurement should be closely related to the intervention under evaluation, especially in a longitudinal study.

## Competing interests

The authors declare that they have no competing interests.

## Authors' contributions

IJH was principal investigator and won funding for the study, CB and IJH were responsible for the design of the study, ethics and management; IJH, SB and MG supervised the study, CB and RK collected data. CB analysed the data under supervision of IJH. CB drafted the paper with input from IJH. All authors commented on and critically revised the paper. CB and IJH are co-guarantors.

## Pre-publication history

The pre-publication history for this paper can be accessed here:

http://www.biomedcentral.com/1472-684X/9/22/prepub

## References

[B1] Gysels M Higginson IJ Access to services for patients with chronic obstructive pulmonary disease: the invisibility of breathlessness J Pain Symptom Manage 2008 36 5 451 60 10.1016/j.jpainsymman.2007.11.008 18495412

[B2] Booth S Moosavi SH Higginson IJ The etiology and management of intractable breathlessness in patients with advanced cancer: a systematic review of pharmacological therapy Nat Clin Pract Oncol 2008 5 2 90 100 10.1038/ncponc1034 18235441

[B3] Bausewein C Booth S Gysels M Higginson I Non-pharmacological interventions for breathlessness in advanced stages of malignant and non-malignant diseases Cochrane Database Syst Rev 2008 2 CD005623 18425927 10.1002/14651858.CD005623.pub2

[B4] Schwartzstein RM Lahive K Pope A Weinberger SE Weiss JW Cold facial stimulation reduces breathlessness induced in normal subjects American Review of Respiratory Disease 1987 136 1 58 61 3605841 10.1164/ajrccm/136.1.58

[B5] Baltzan M Fan to palliate exercise-induced dyspnea with severe COPD [abstract] American Journal of Respiratory and Critical Care Medicine 2000 161 3 Suppl A59

[B6] Galbraith S Fagan P Perkins P Lynch A Booth S Does the use of a handheld fan improve intractable breathlessness? Journal of Pain & Symptom Management 2009 2009 in press 10.1016/j.jpainsymman.2009.09.024 20471544

[B7] Bausewein C Booth S Gysels M Kuhnbach R Haberland B Higginson IJ Individual breathlessness trajectories do not match summary trajectories in advanced cancer and chronic obstructive pulmonary disease: results from a longitudinal study Palliat Med 2010 in press 20847087 10.1177/0269216310378785

[B8] GOLD, Global Initiative for Chronic Obstructive Lung Disease Global strategy for the diagnosis, management and prevention of chronic obstructive lung disease; updated report 2007

[B9] Pocock S Clincial trials A practical approach 1983 Chichester: John Wiley & Sons

[B10] Borg GA Psychophysical bases of perceived exertion Med Sci Sports Exerc 1982 14 5 377 81 7154893

[B11] Burdon JGW Juniper EF Killian KJ Hargreave FE Campbell EJM The perception of breathlessness in asthma American Review of Respiratory Disease 1982 126 825 8 7149447 10.1164/arrd.1982.126.5.825

[B12] Dorman S Jolley C Abernethy A Currow D Johnson M Farquhar M Researching breathlessness in palliative care: consensus statement of the National Cancer Research Institute Palliative Care Breathlessness Subgroup Palliat Med 2009 23 3 213 27 10.1177/0269216309102520 19251835

[B13] Booth S Kelly MJ Cox NP Adams L Guz A Does oxygen help dyspnea in patients with cancer? Am J Respir Crit Care Med 1996 153 5 1515 8 8630595 10.1164/ajrccm.153.5.8630595

[B14] Cohen J Statistical power analysis for the behavioral sciences 1988 Second Hillsdale: Lawrence Earlbaum Associates

[B15] White C Hardy J Gatekeeping from palliative care research Progress in Palliative Care 2008 16 4 167 71 10.1179/096992608X346189

[B16] King M Nazareth I Lampe F Bower P Chandler M Morou M Impact of participant and physician intervention preferences on randomized trials: a systematic review JAMA 2005 293 9 1089 99 10.1001/jama.293.9.1089 15741531

[B17] Preference Collaborative Review Group Patients' preferences within randomised trials: systematic review and patient level meta-analysis BMJ 2008 337 a1864 10.1136/bmj.a1864 18977792 PMC2659956

[B18] Mann H Djulbegovic B Choosing a control intervention for a randomised clinical trial BMC Med Res Methodol 2003 3 7 10.1186/1471-2288-3-7 12709266 PMC165581

[B19] American Thoracic Society Dyspnea: Mechanisms, Assessment and Management: A Consensus Statement Am J Resp Crit Care Med 1999 159 321 40 9872857 10.1164/ajrccm.159.1.ats898

